# Link between the unfolded protein response and dysregulation of mitochondrial bioenergetics in Alzheimer’s disease

**DOI:** 10.1007/s00018-019-03009-4

**Published:** 2019-01-25

**Authors:** Yannik Poirier, Amandine Grimm, Karen Schmitt, Anne Eckert

**Affiliations:** 1 Transfaculty Research Platform, Molecular and Cognitive Neuroscience, University of Basel, Basel, Switzerland; 2 Neurobiology Laboratory for Brain Aging and Mental Health, Psychiatric University Clinics Basel, Basel, Switzerland

**Keywords:** ER stress, Unfolded protein response, Mitochondrial dysfunction, Alzheimer’s disease, Amyloid-β peptide, Tau protein

## Abstract

Alzheimer’s disease (AD) is a progressive neurodegenerative disorder affecting more than 47.5 million people worldwide. Metabolic impairments are common hallmarks of AD, and amyloid-β (Aβ) peptide and hyperphosphorylated tau protein—the two foremost histopathological signs of AD—have been implicated in mitochondrial dysfunction. Many neurodegenerative disorders, including AD, show excessive amounts of mis-/unfolded proteins leading to an activation of the unfolded protein response (UPR). In the present study, we aimed to characterize the link between ER stress and bioenergetics defects under normal condition (human SH-SY5Y neuroblastoma cells: control cells) or under pathological AD condition [SH-SY5Y cells overexpressing either the human amyloid precursor protein (APP) or mutant tau (P301L)]. More specifically, we measured UPR gene expression, cell viability, and bioenergetics parameters, such as ATP production and mitochondrial membrane potential (MMP) in basal condition and after an induced ER stress by thapsigargin. We detected highly activated UPR and dysregulated bioenergetics in basal condition in both AD cellular models. Strikingly, acute-induced ER stress increased the activity of the UPR in both AD cellular models, leading to up-regulation of apoptotic pathways, and further dysregulated mitochondrial function.

**Electronic supplementary material:**

The online version of this article (10.1007/s00018-019-03009-4) contains supplementary material, which is available to authorized users.

## Introduction

AD is a progressive neurodegenerative disorder affecting more than 47.5 million people worldwide and will certainly gain more attention in the coming years [1, 2]. There are two types of AD: the sporadic form and the familial form. The sporadic form is the most common type and patients show their first behavioural abnormalities starting at 65 years [3]. The cause of the sporadic form remains unknown. However, they are few cases (1–6%), where the onset of the disease starts at early age (between 35 and 65 years) [3]. Early onset cases are often genetically inherited and, therefore, termed familial AD (FAD). As the late onset, the early onset is driven by selectively declarative memory loss, followed by fading of cognitive abilities such as language skills, problem solving, and visuospatial perception [4, 5]. From a histopathological point of view, AD is characterized by the presence of amyloid-β (Aβ) plaques and neurofibrillary tangles (NFTs) formed by abnormally tau hyperphosphorylation in the brain. Mutations in amyloid precursor protein (APP), the principal component of Aβ plaques and presenilin 1/2, were linked to FAD in genetic studies [6]. Interestingly, no mutation in the tau-encoding genes was linked to FAD. However, mutations of tau were found in another form of inherited dementia, namely, frontotemporal dementia with Parkinsonism linked to chromosome 17 (FTDP-17). It shares features such as memory loss or language impairments with AD [7], but it lacks plaques [8].

Mutation in both genes was linked to mitochondrial deficits at an early AD stage [9, 10] as well as to an activated UPR [11, 12]. Indeed, metabolic defects and mitochondrial dysfunction have been demonstrated to be one of the mechanisms underlying Aβ-induced neurodegeneration [13], and our research group already showed that the two hallmark proteins, Aβ peptide (forming amyloid plaques), and abnormally hyperphosphorylated tau protein (aggregating in NFTs) are involved in these processes [10, 14]. Furthermore, recent studies present ER stress, as a common feature of AD [11, 15, 16]. Many neurodegenerative disorders, including AD, show excessive amounts of mis-/unfolded proteins leading to an activation of the UPR. Upon ER stress, the UPR tries to feedback control the stress by an adaptive response, up-/down-regulating genes, which regulate cell fate [17, 18]. However, whether ER stress is a causative factor or a consequence of AD pathology is still under debate.

In the present study, we investigated the effect of overexpression of the two AD hallmark proteins, amyloid-β and hyperphosphorylated tau, on the activity of the UPR, bioenergetics, and cell viability in basal condition and after acute ER stress induced by thapsigargin in the following cellular models of AD: SH-SY5Y neuroblastoma cells overexpressing amyloid precursor protein (APP) leading to increased Aβ or the mutant form of tau protein (P301L) leading to hyperphosphorylated tau, compared to control cells (empty vector transfected cells = mock cells and expressing the wild-type form of tau protein = WT Tau cells, respectively). Thapsigargin (Th), which irreversibly inhibits the sarcoplasmic/endoplasmic reticulum Ca^2+^-ATPase (SERCA) [19], was used to induce an ER stress. Since metabolic disturbances are strongly associated with increased risk for AD and are a potent inducer of the UPR [20], activation of the UPR and mitochondrial function was characterized after an acute Th-induced ER stress in Aβ and tau-overexpressing cells. In particular, we profiled whether our Aβ- and tau-overexpressing cellular models showed activated UPR and dysregulated mitochondrial bioenergetics in basal condition and what were the effects of an additional ER stress in our AD cellular models.

## Materials and methods

### Chemicals and reagents

Dulbecco’s-modified Eagle medium (DMEM), fetal calf serum (FCS), penicillin/streptomycin, Cell Proliferation Kit I (MTT), Cytotoxicity Detection Kit (LDH), TMRM, neurobasal medium, and retionic acid were from Sigma-Aldrich (St. Louis, MO, USA). Glutamax, B27 supplement and AlexaFluor 568 goat anti-mouse IgG (H + L) were from Gibco Invitrogen (Waltham, MA, USA). Horse serum (HS) was from Amimed, Bioconcept (Allschwil, Switzerland). Thapsigargin was from Santa Cruz Tech. (TX, USA). ViaLight plus kit (ATP) was from Lonza (BS, Switzerland). RT^2^ First Strand Kit was from QIAGEN (Hilden, NW, Germany). NGF was from Lubio (Zürich, Switzerland). Collagen I and Phospho-Tau (Ser202, Thr205) Monoclonal Antibody (AT8) were from Thermo fisher. Okadaic acid was from Adipogen.

### Cell culture

Human neuroblastoma SH-SY5Y cells stably expressing vector alone (pCEP4, control cells: Mock cells) or the entire coding region of human wild-type APP (APP695, APP cells) were used. Stably transfected APP cells were selected regularly using hygromycin (300 µg/ml) [21] and checked on a routine basis for APP expression levels [14]. In addition, human neuroblastoma SH-SY5Y cells stably transfected with human wild-type tau (Htau40, WT Tau cells) and P301L mutant tau (P301L cells) were used. Both WT Tau and P301L cells showed similar tau expression levels [22, 23]; however, the P301L mutation is required for abnormal tau hyperphosphorylation and filament formation [23]. Cells were regularly selected with G418 (300 µg/ml) and screened on a regular basis by routine histochemical and biochemical assays. Cells were cultured in Dulbecco’s-modified Eagle’s medium (DMEM) with additionally 1% penicillin/streptomycin/Glutamax (PSG) 10% heat-inactivated fetal calf serum (FCS) at 37 °C in a humidified incubator chamber under 7.5% CO_2_ atmosphere. The cells were kept in culture and in 10 cm^2^ dishes, split twice a week, and plated when they reached around 80% confluence, 1 day prior the treatment. In experiments using differentiated cells, Mock, APP, WT Tau, and P301L cells were plated at a density of 5 × 10^4^ cells/well. Cells were grown in neurobasal medium supplemented with 2% B27, 2 mM Glutamax, and 1% (v/v) penicillin/streptomycin. Cells were treated for 5 days with 10 uM of retinoic acid and 50 ng/ml of NGF (nerve growth factor) before the treatment with thapsigargin.

### Treatment paradigm

To measure the toxicity of thapsigargin on neuroblastoma cells, assessments of cell toxicity (LDH) were first performed on Mock cells to determine the non-toxic concentration of Th (10–10,000 nM, data not shown) and after different treatment durations, acute (3H), and chronic (24H), using LDH release assay. Cells were seeded in white (clear bottom) 96-well plates at a density of 1.5 × 10^5^ cells/well and the LDH assay was performed according to the manufacturer’s instructions. On the basis of the LDH results, and according to the previous data obtained in Mock cells (PMID: 25657664, PMID: 25581772), the concentration of thapsigargin of 500 nM was then selected and used in all assays for all cell types. Cells were treated 1 day after plating for acute or chronic treatment with the selected concentration.

### Determination of ATP levels

Cells were plated into 96-well cell culture plates at a density of 1.5 × 10^5^ cells/well. Total ATP content of basal (no treatment) and Th-treated neuroblastoma cells was measured using a bioluminescence assay (ViaLight plus kit) according to the instruction of the manufacturer. The bioluminescent method measures the formation of ATP by luciferase. The emitted light is linearly proportional to the ATP concentration and was measured using the Cytation 3 Cell Imaging Multi-mode Reader (BioTek).

### Mitochondrial membrane potential (MMP)

Cells were plated into a black 96-well plate at a density of 1.5 × 10^5^ cells/well. The MMP was measured using the fluorescent dye tetramethylrhodamine methyl ester perchlorate (TMRM). Transmembrane distribution of the dye is dependent on MMP. Cells were loaded with the dye at a concentration of 0.4 µM for 15 min at room temperature in the dark on an orbital shaker. After washing twice with HBSS, the fluorescence was detected using the Cytation 3 cell Imaging Multi-mode Reader (BioTek) at 531 nm (excitation)/595 nm (emission).

### Quantitative real-time PCR

Cells were plated in clear 6-well plates at a density of 2.5 × 10^5^ cells/well in DMEM medium containing 10% FCS, 1% PSG. Total RNA was extracted from lysates of basal (non-treated) and Th-treated cells using the RNeasy Mini Kit (QIAGEN) and the RNA concentration was assessed with a Nanodrop 1000 spectrophotometer. cDNA was generated using the RT^2^ First Strand Kit (QIAGEN) and performed according to the instruction of the manufacturer. Briefly, cDNA was added to RT^2^ Profiler Green Mastermix and loaded in 96-well plates (taqman gene array plate), which were pre-coated with primers specific for the human unfolded protein response provided by the company. Quantitative real-time PCR was performed using Step One Plus system (Applied Biosystems). The CT values (threshold cycle: number of cycle until sample signal is detected) were exported and analyzed with the GeneGlobe Data Analysis Centre Software on the website of Qiagen: https://www.qiagen.com/us/shop/genes-and-pathways/data-analysis-center-overview-page/?akamai-feo=off.

### Immunocytochemistry

Cells were plated at a density of 4 × 10^5^ cells on collagen-treated coverslips. After 2 days growing, cells were treated with 500 nM thapsigargin or 100 nM okadaic acid for 3 h. Coverslips were fixed in PBS with 10% formaldehyde for 10 min and then permeabilized with 0.1% Triton for 15 min and blocked with 2% BSA for 30 min at RT. The coverslips were incubated overnight at 4 °C with the primary antibody [Phospho-Tau (Ser202, Thr205) Monoclonal Antibody (AT8), Thermo Fisher; 1:250]. After washing with PBS, they were incubated for 1 h at RT with the secondary antibody (AlexaFluor 568 goat anti-mouse IgG, Thermo Fisher, Switzerland, 1:500). Negative controls, where the primary antibody was omitted, were included in all experiments. The acquisition of XY-confocal images with maximum intensity projection was performed using an inverted microscope (Leica Microsystems TCS SPE DMI4000) attached to an external light source for enhanced fluorescence imaging (Leica EL6000) with a × 63 oil immersion objective (numerical aperture 1.4; Leica). Pinhole settings were chosen in such a way that axially each cell was entirely present within the confocal volume. For morphological analysis, the samples were blindly analyzed. The fluorescence intensity of phospho-tau was measured for each cell using the NIH ImageJ software. Briefly, individual cells were outlined, the background value was subtracted, and fluorescence intensity was taken as average pixel intensity over the total cell area.

### Statistical analysis

Statistical Analysis was performed with the Graph Pad Prism software. Data were presented as mean ± SEM and normalized to the corresponding non-treated control group or to the Mock non-treated cells (= 100%). For the comparisons of two groups, student unpaired *t* test was used and for the comparison of more than two groups, One-way ANOVA was used, followed by a Turkey’s Multiple Comparison Test. *P* values ≤ 0.05 = *, *P* ≤ 0.01 = **, and *P* ≤ 0.001 = *** were considered statistically significant.

## Results

### APP/Aβ and tau-overexpressing cells express an activated UPR

**Fig. 1:**
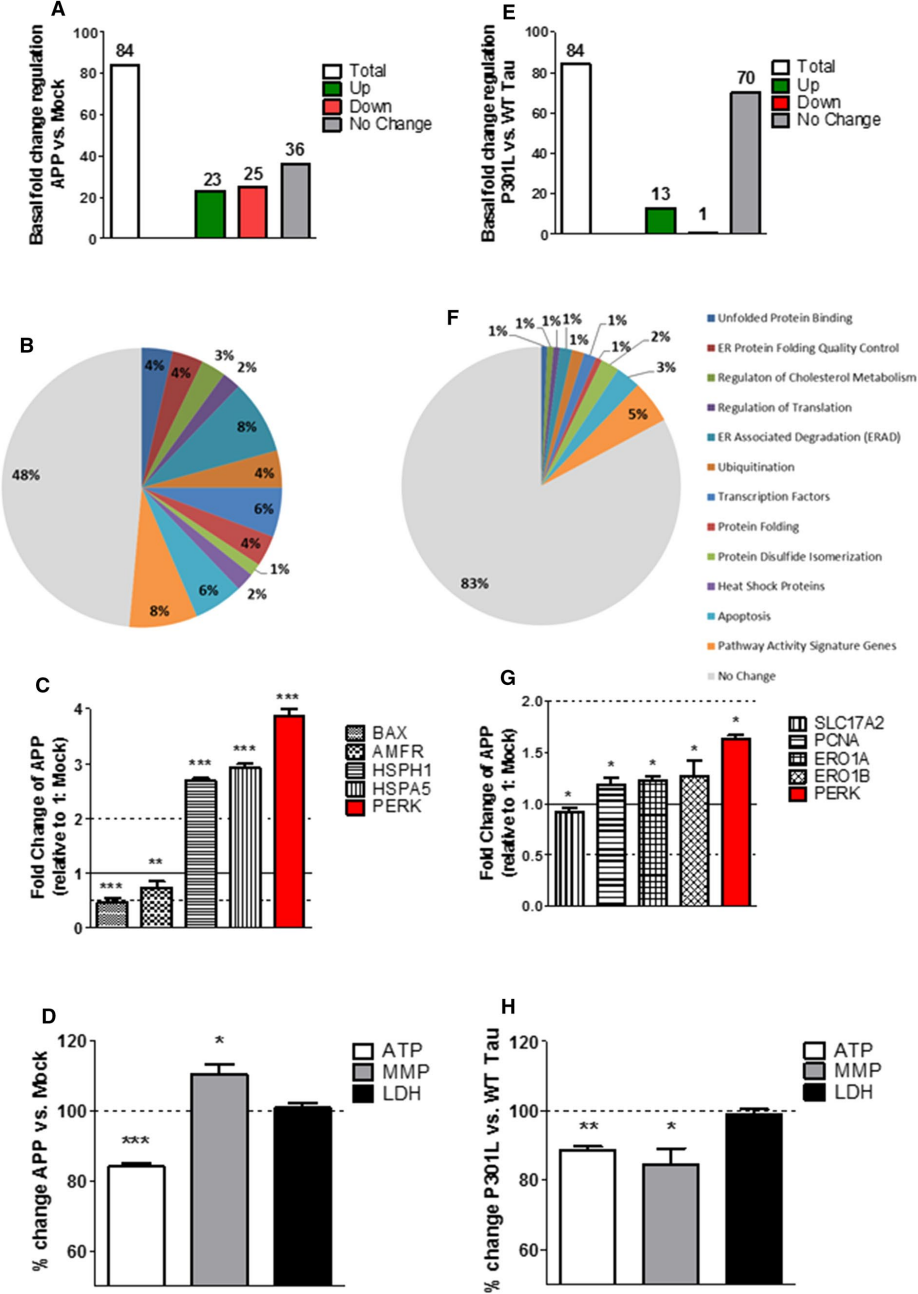
Regulation of UPR gene expression, mitochondrial bioenergetics, and cell viability in basal condition in APP and P301L cells. **a**, **e** Basal (non-treated) fold-change regulation of UPR genes in **a** APP cells compared to Mock cells and **E** P301L cells compared to WT Tau cells. **b**, **f** Pie charts of significantly up-/down-regulated UPR genes (colored) and the non-changed UPR genes (grey) sorted in groups by pathways in basal condition in **b** APP cells compared to Mock cells and **f** P301L cells compared to WT Tau cells. Of note, the total number of genes measured is 84; as several genes are involved in diverse pathways, the pie chart is composed of total 140 genes (see Suppl. Table 4). **c**, **g** Selection of significantly up-/down-regulated UPR genes in **c** APP cells and **g** P301L cells. Fold-change values greater than one indicate a positive- or an up-regulation, values less than one indicate a negative or a down-regulation. **a**–**c**, **e**–**g** Values were calculated based on a Student’s *t* test of the replicate $2^{{ - \Delta {\text{Ct}}}}$ values for each gene (*n* = 3 replicates of 3 independent experiments), and *P* < 0.05 were considered significant. **d**, **h** Basal ATP level, MMP level, and LDH level in **d** APP cells compared to Mock cells and (H) P301L cells compared to WT tau cells. Values represent the mean ± SEM (*n* = 18–60 replicates of 3–5 independent experiments) and were normalized to non-treated **d** Mock cells and **h** WT Tau cells (= 100%). Statistical analysis was performed using One-Way ANOVA followed by Turkey’s Multiple Comparison Test: *P < 0.05, ** *P* < 0.01 and ***P < 0.001. *ATP* adenosine triphosphate (major energy source of cells), *MMP* mitochondrial membrane potential (indicator of polarization state of the mitochondrial membrane), *LDH* lactate dehydrogenase (released by cells into medium when integrity of cell membrane is lost—cytotoxicity detection)

To assess whether the presence of two hallmark proteins of AD, APP/Aβ, or tau have per se an influence on the UPR, the regulation of gene expression activity of 84 UPR genes was measured in Mock, APP, P301L, and WT Tau cells using quantitative real-time PCR. Both APP (Fig. 1a) and P301L cells (Fig. 1e) showed an activated UPR compared to basal Mock and WT Tau cells, respectively, as several genes were significantly up-/down-regulated including a key UPR initiator: PERK (Fig. 1c, g). On the 84 genes tested, 23 genes were significantly up-regulated, 25 significantly down-regulated, and 36 were not significantly changed in APP cells (Fig. 1a). The significantly up-/down-regulated genes were involved in both pro-cell-survival and pro-apoptotic pathways (Fig. 1b; Suppl. Table 1). However, APP cells seem to maintain the UPR in a harmless range by significantly up-regulating pro-cell-survival genes such as HSPA5 and HSPH1, while the pro-cell-apoptotic genes BAX and AMFR were significantly down-regulated (Fig. 1c). P301L cells on the other hand significantly up-regulated 13 genes and only 1 gene was significantly down-regulated, while 70 genes showed no significant changes (Fig. 1e). The significantly up-regulated genes were involved in both pro-cell-survival and pro-apoptotic pathways; however, the fold change was rather small, since no genes had a fold change less than 0.5 or higher than 2 (Fig. 1f; Suppl. Table 3). Furthermore, they also showed significantly up-regulated pro-cell-survival genes such as ERO1A, ERO1B, and PCNA (Fig. 1g). The only significantly down-regulated gene was SLC17A2, which is a pathway activity signature gene and was shown to code for a protein regulating membrane potential [24]. In addition, no significant activation of the UPR was measured in WT Tau cells (compared to Mock cells) (Suppl. Figure 1). Taken together, these results confirm that APP/Aβ and hyperphosphorylated tau exhibit an activated UPR. Moreover, the UPR seems to be more activated in APP/Aβ than in the tau-overexpressing cells, since more genes have a significant fold change and the increase (over 2) and the decrease (under 0.5) of the fold change is higher.

### Mitochondrial bioenergetics is differently impaired in APP/Aβ and tau-overexpressing cells

To determine the influence of APP/Aβ or hyperphosphorylated tau on mitochondrial bioenergetics and cell viability, mitochondrial parameters such as ATP: adenosine triphosphate (major energy source of cells); MMP: mitochondrial membrane potential (indicator of polarization state of the mitochondrial membrane) and LDH: lactate dehydrogenase (released by cells into medium when integrity of cell membrane is lost = cytotoxicity detection) were measured under basal condition in all four cell types (Mock, APP, P301L, and WT Tau). APP cells showed a significant decrease of ATP level (– 16% compared to Mock cells) and a slight increase of the MMP (+ 10% compared to Mock cells) translating to a hyperpolarization of the mitochondrial membrane potential (Fig. 1d). Nevertheless, no change in LDH level was measured indicating that APP/Aβ overexpressing cells did not induce any cell toxicity compared to Mock cells (Fig. 1d). The same experiment was performed in WT Tau and P301L cells, to assess the effect of mutant tau. ATP level was also significantly decreased (– 12% compared to WT Tau cells), which was paralleled by a reduction of the MMP (**–** 16% compared to WT Tau cells) revealing a depolarization of the mitochondrial membrane potential (Fig. 1h). Furthermore, no significant change in LDH level was measured, indicating that no cell toxicity induced by overexpression of mutant tau compared to WT tau cells (Fig. 1h). Nearly, identical results of dysregulated bioenergetics were obtained in differentiated cells (Suppl. Figure 2) and are, therefore, independent of the differentiation state of the cell. Taken together, these results indicate a dysregulation of mitochondrial bioenergetics in APP/Aβ and tau-overexpressing cells. Nevertheless, no cell toxicity was detected.

### Acute thapsigargin-induced ER stress increases the UPR and mitochondrial dysfunction in APP/Aβ overexpressing cells

**Fig. 2:**
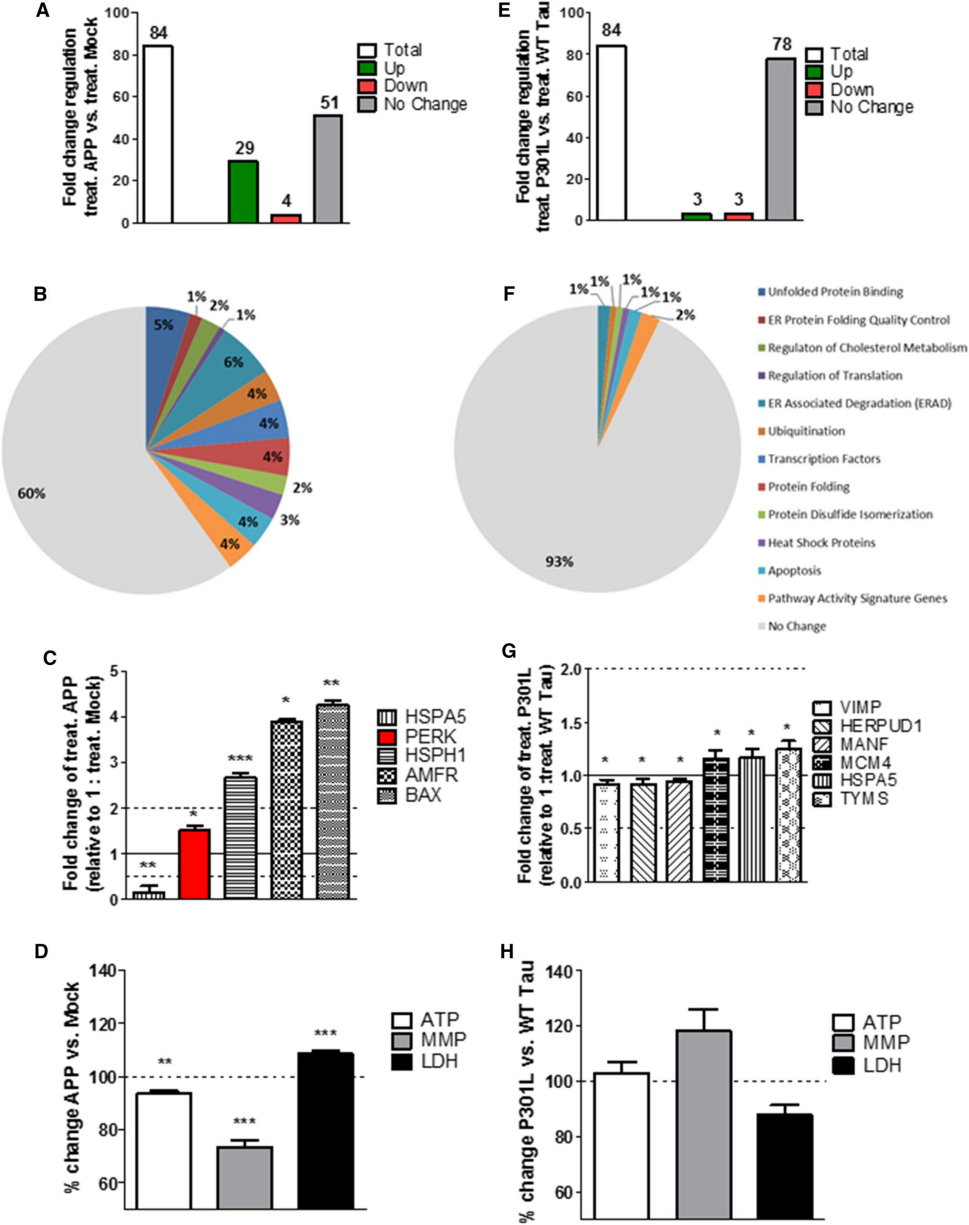
Regulation of UPR genes expression, mitochondrial bioenergetics, and cell viability after acute thapsigargin (Th) treatment in APP and P301L cells. **a**, **e** Fold-change regulation of UPR genes in (**a**) Th-treated APP cells compared to Th-treated Mock cells and **e** Th-treated P301L cells compared to Th-treated WT Tau cells. **b**, **f** Pie charts of significantly up-/down-regulated UPR genes (colored) and the non-changed UPR genes (grey) sorted by their function, in acute ER stress condition of **b** Th-treated APP cells compared to Th-treated Mock cells and **f** Th-treated P301L cells compared to Th-treated WT Tau cells. Of note, the total number of genes measured is 84; as several genes are involved in diverse pathways, the pie chart is composed of total 140 genes (see Suppl. Table 4). **c**, **g** Selection of significantly up-/down-regulated UPR genes by fold regulation in **c** Th-treated APP cells and **g** Th-treated P301L cells. Fold-change values greater than one indicate a positive- or an up-regulation, values less than one indicate a negative or down-regulation. **a**–**c**, **e**–**g** Values were calculated based on a Student’s *t* test of the replicate $2^{{ - \Delta {\text{Ct}}}}$ values for each gene (*n* = 3 replicates of 3 independent experiments), and *P* < 0.05 were considered significant. **d**, **h** ATP level, MMP level, and LDH level of **d** Th-treated APP cells compared to Th-treated Mock cells and **h** Th-treated P301L cells compared to Th-treated WT tau cells. Values represent the mean ± SEM (*n* = 18–60 replicates of 3–5 independent experiments) and were normalized to Th-treated (D) Mock cells and **h** WT Tau cells (= 100%). Statistical analysis was performed using One-Way ANOVA followed by Turkey’s Multiple Comparison Test: **P < 0.01 and ***P < 0.001. *ATP* adenosine triphosphate (major energy source of cells), *MMP* mitochondrial membrane potential (indicator of polarization state of the mitochondrial membrane), *LDH* lactate dehydrogenase (released by cells into medium when integrity of cell membrane is lost—cytotoxicity detection)

Next, the acute effect of Th-induced ER stress on the UPR, the mitochondrial bioenergetics, and cell viability of APP/Aβ and tau-overexpressing cells was measured by applying thapsigargin (Th), a frequently used ER stressor, for 3 h. Acute thapsigargin treatment induced a stronger unfolded protein response in APP cells compared to Th-treated Mock cells represented by higher fold changes in gene expression (Suppl. Table 1). On the 84 genes tested, 29 genes were significantly up-regulated, 4 significantly down-regulated, and 51 had no significant fold change (Fig. 2a). Moreover, the fold change of the significantly up-/down-regulated genes increased, with 4 genes that were down-regulated less than 0.5-fold and 20 genes that were up-regulated more than 2-fold (Suppl. Table 1). However, the activated genes were still up-regulated in both pro-cell-survival and pro-cell-apoptotic pathways. Specifically, 13 up-regulated genes are involved in ER-associated degradation (ERAD) and apoptotic pathways and 10 up-regulated genes are involved in protein folding, binding, and quality control (Fig. 2b, Suppl. Table 1). Moreover, the fold change increased more in the Th-treated APP cells (compared to the Th-treated Mock cells) than in untreated APP cells (compared to basal Mock cells) (Suppl. Table 1). One of the UPR key initiator, PERK, was still up-regulated and the pro-cell survival gene HSPA5 which was up-regulated in the basal condition was now down-regulated after ER stress (Fig. 2c). HSPH1 stayed up-regulated, whereas pro-apoptotic genes such as AMFR and BAX which were down-regulated in basal condition were up-regulated after ER stress (Fig. 2c). To characterize the mitochondrial dysregulation in terms of bioenergetics and cell viability after an acute ER stress on APP/Aβ-overexpressing cells, acute Th-treated APP cells were compared to Th-treated Mock cells. Even if the Th treatment per se induced a significant increase of ATP and MMP level in both Mock and APP cells (compared to the non-treated cells, respectively) (data not shown), the presence of APP/Aβ overexpression induced a significant decrease of ATP level (**–** 17% compared to Th-treated Mock cells) and MMP level (– 27% compared to Th-treated Mock cells) in Th-treated APP cells (Fig. 2d). Nearly, identical results of dysregulated bioenergetics were obtained in differentiated cells (Suppl. Figure 2) and are, therefore, independent of the differentiation state of the cell. Furthermore, LDH level was increased (**+** 9% compared to Th-treated Mock cells) indicating cell death (Fig. 2d).

### Acute ER stress in mutant tau-overexpressing cells induces almost no change in UPR genes expression and mitochondrial bioenergetics

When comparing Th-treated WT Tau and P301L cells, of note, ER stress seemed to have almost no effect on the fold change of UPR genes. From the total 84 genes tested, only 3 genes were significantly up- and down-regulated (Fig. 2e). The 3 significantly up-regulated genes were HSPA5 coding for a heat shock protein and MCM4 and TYMS which are pathway activity signature genes (Fig. 2f, g). The three down-regulated genes were VIMP, HERPUD1, and MANF which are involved in ERAD, ubiquitination, and apoptotic pathways. However, the fold change was only slightly changed in P301L cells (compared to Th-treated WT Tau cells) (Fig. 2f, g) and PERK, the major UPR initiator was not significantly activated. In line with the weak UPR up-regulation, no significant change in cell viability and mitochondrial bioenergetics of undifferentiated (Fig. 2h) and differentiated cells (Suppl. Figure 2) was measured.

### WT Tau and P301L cells show identical up-regulation of UPR genes and dysfunction of mitochondrial bioenergetics after acute ER stress

**Fig. 3:**
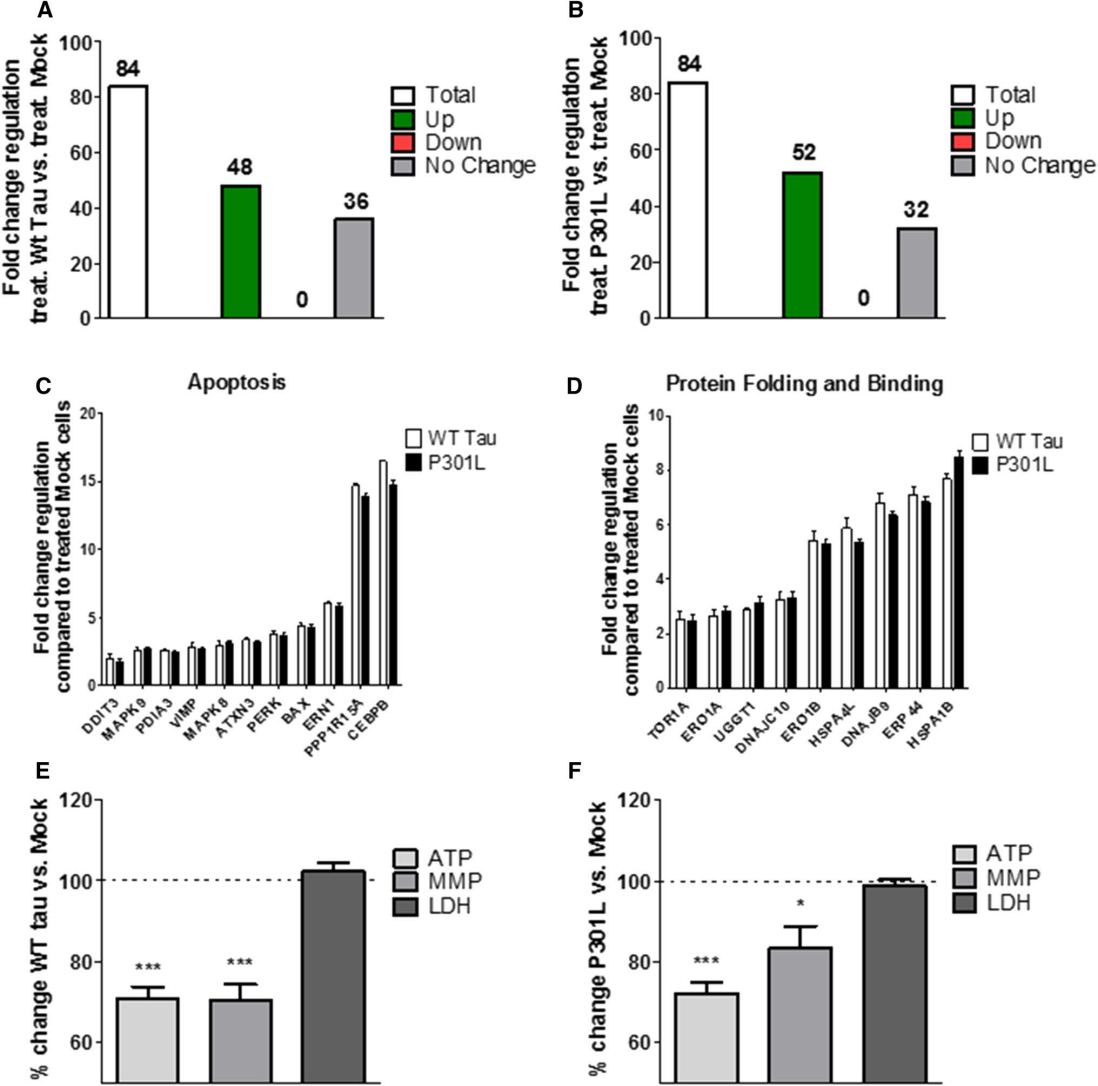
Regulation of UPR genes expression, bioenergetics, and cell viability of thapsigargin-treated **a** WT Tau and **b** P301L cells compared to thapsigargin-treated Mock cells. **a**, **b** Fold-change regulation of UPR genes of Th-treated **a** WT Tau cells and **b** P301L cells compared to Th-treated Mock cells. (**c**, **d**) Fold-change regulation of significantly up-regulated UPR genes in Th-treated WT Tau and P301L cells compared to Th-treated Mock cells of two selected functional groups. Values were calculated based on a Student’s *t* test of the replicate $2^{{ - \Delta {\text{Ct}}}}$ values for each gene (*n* = 3 replicates of 3 independent experiments), and *P* < 0.05 were considered significant. **e**, **f** ATP level, MMP level and LDH level of **e** Th-treated WT Tau cells and **f** Th-treated P301L cells compared to Th-treated Mock cells. Values represent the mean ± SEM (*n* = 18–60 replicates of 3–5 independent experiments) and were normalized to Th-treated Mock cells (= 100%). Statistical analysis was performed using One-Way ANOVA followed by Turkey’s Multiple Comparison Test: *P < 0.05 and ***P < 0.001. *ATP* adenosine triphosphate (major energy source of cells), *MMP* mitochondrial membrane potential (indicator of polarization state of the mitochondrial membrane), *LDH* lactate dehydrogenase (released by cells into medium when integrity of cell membrane is lost—cytotoxicity detection)

To further understand the effects of the presence of WT Tau and P301L-tau on the UPR and bioenergetics in ER stress conditions, we compared them to the control cells Mock. Both, WT Tau cells (Fig. 3a) and P301L cells (Fig. 3b) showed a significant activation of the UPR after acute Th treatment by up-regulating 48 genes (WT Tau cells) and 52 genes (P301L cells) when compared to Th-treated Mock cells. Interestingly, the acute ER stress seemed to have the same effect on both, WT Tau and P301L cells, since they both showed a significant increase in the expression of 46 identical genes (Suppl. Table 3). Interestingly, the up-regulated genes also displayed the same level of the fold change, demonstrating the identical effect of the ER stress on the two different cell types (Fig. 3c). To display the effect of the induced ER stress on the mitochondrial dysfunction in WT Tau and P301L cells, the same mitochondrial parameters as described previously were compared to Th-treated Mock cells. In line with the identical effect on the UPR, mitochondrial bioenergetics was decreased significantly in both cell types. WT Tau cells showed a decrease of ATP level (– 29% compared to Th-treated Mock cells) paralleled by a depolarization of the membrane potential (– 30% compared to Th-treated Mock cells) (Fig. 3e). P301L cells also decreased ATP level (– 28% compared to Th-treated Mock cells) and MMP levels (– 17% compared to Th-treated Mock cells) (Fig. 3f). However, the cell viability seemed not to be affected after an acute treatment, since no significant change in LDH level was measured (Fig. 3e, f). Taken together, WT Tau and P301L cells seem to respond almost identical to an acute-induced ER stress by thapsigargin, by up-regulating the same genes of the UPR and showing a similar decrease of bioenergetics when compared to Th-treated Mock cells.

### WT Tau cells express similar high levels of phospho-tau as P301L cells after acute thapsigargin treatment

Since WT Tau and P301L cells show identical up-regulation of UPR genes and dysfunction of mitochondrial bioenergetics after acute thapsigargin treatment, fluorescence intensity of AT8 antibody, which detects specific epitopes of phospho-tau (Ser202, Thr205) that have been widely suggest to contribute in AD progression, was measured by immunocytochemistry under the confocal microscopy. While WT Tau cells showed almost no phospho-tau signal in basal condition, P301L cells expressed high level of phospho-tau. However, acute thapsigargin treatment increased expression of phospho-tau in both WT Tau and P301L cells to an almost identical level. In addition, treatment with okadaic acid was performed to identify maximal level of phospho-tau expression (Supp. Figure 3)

## Discussion

**Fig. 4:**
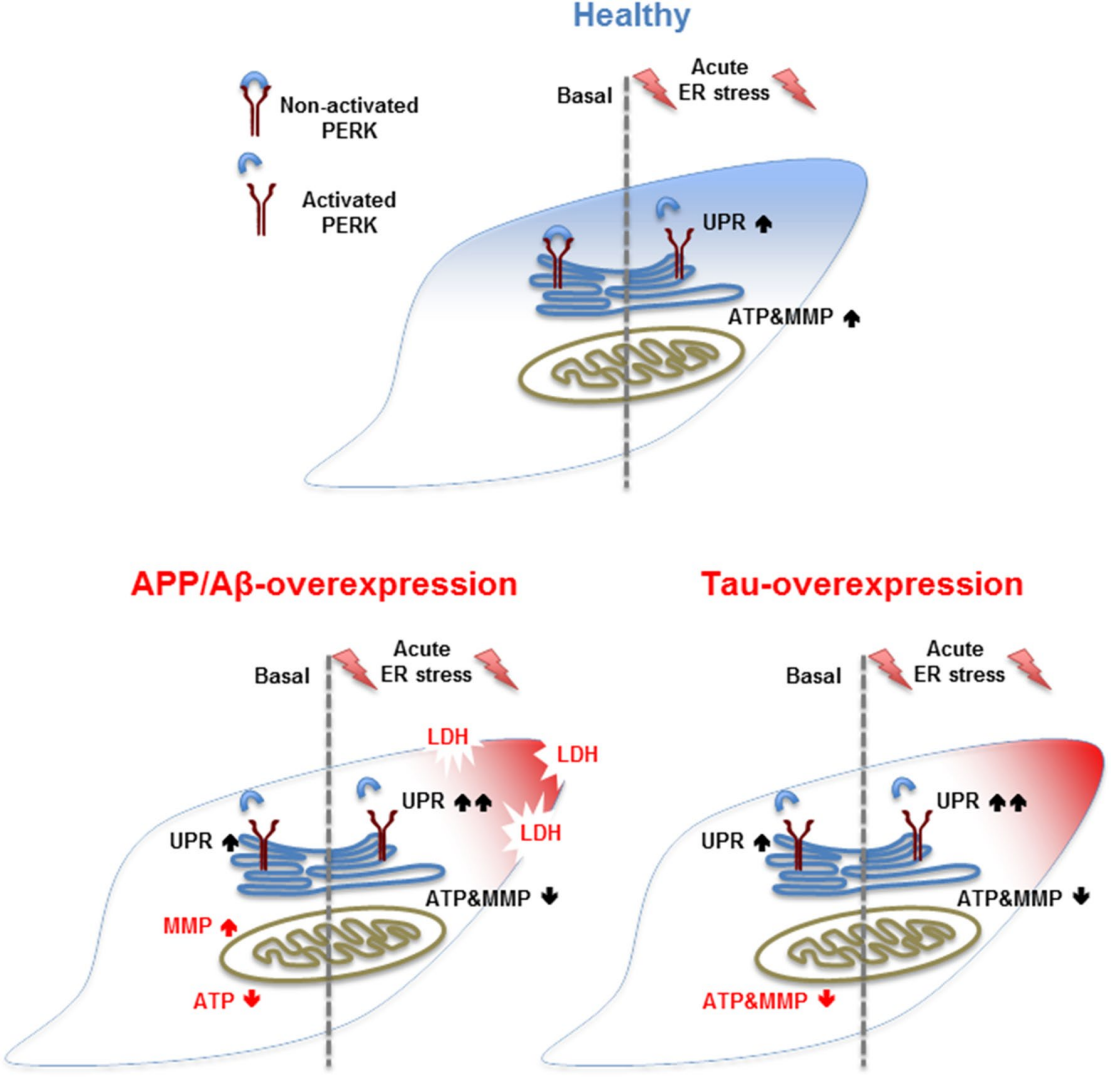
Summary of the findings. (Blue) In basal condition, healthy cells show no activated UPR and no mitochondrial dysregulation, whereas an acute-induced ER stress by Th activates the UPR and increases MMP and ATP level. (Red) Both AD model show an activated UPR in basal condition and differently impaired mitochondrial function, such as MMP and ATP level. An additional acute Th-induced ER stress further activates the UPR and decreases MMP and ATP level in both tau-overexpressing cells; however, APP/Aβ-overexpressing cells already show some cell toxicity, displayed by increased LDH level

Many neurodegenerative diseases, such as AD, have been associated with ER stress and mitochondrial dysfunction [9, 25–27]. In the present study, we distinguished the activation of the unfolded protein response (UPR) and parameters of mitochondrial function, such as bioenergetics and the cell viability in APP/Aβ and tau-overexpressing cells in basal condition and after acute thapsigargin-induced ER stress. Our key findings were that (1) APP/Aβ and tau-overexpressing cells display already an activated UPR and differently impaired mitochondrial function in basal condition. Moreover, (2) APP/Aβ and tau-overexpressing cells further increase the UPR and still show differently impaired mitochondrial dysfunction after acute thapsigargin-induced ER stress, with APP/Aβ-overexpressing cells being more vulnerable by showing cell toxicity already after 3 h. Finally, (3) whereas the presence of mutant tau seem to have a significant effect on the UPR and the mitochondrial dysregulation in basal condition compared to WT Tau, almost no difference on UPR activation and mitochondrial function was observed after an acute-induced Th treatment between the two cell types. However, when both Th-treated cell types were compared to Th-treated Mock cells, the UPR activation was significantly up-regulated almost identically in WT Tau and mutant tau cells. Furthermore, WT Tau cells expressed similar high levels of phospho-tau as P301L cells after acute thapsigargin treatment. Therefore, we suggest a direct functional connection between Th-induced UPR activation and phosphorylation of tau (Fig. 4).

Remarkably, the effect of APP/Aβ and tau-overexpression per se induces UPR activation in basal condition as displayed by significantly up-/down-regulated UPR genes when compared to Mock cells and WT Tau cells, respectively. The UPR transducer PERK, which launches the most immediate response to ER stress [11, 28], was shown to be activated in both AD cell types. These findings are in line with experiments performed on the paternal neuroblastoma cell line SK-N-SH, where it was showed that induction of Aβ triggered the UPR by activating PERK [29]. Another study performed in cases of neuropathologically defined as frontotemporal lobar degeneration with tau pathology (FTLD-tau), linked tau mutation with increased activation of the UPR key initiator pPERK (phosphorylated PERK) [30]. While most studies focus on PERK activation by Aβ, we observed that the overexpression of APP/Aβ induced a significant fold change of many genes involved in both pro-cell-survival and pro-cell-apoptotic pathways. Most genes which were significantly up-regulated are involved in pro-cell-survival pathways, whereas down-regulated genes were found to be involved in pro-apoptotic pathways. Numerous studies describe that upon ER stress the UPR first tries to feedback control the induced stress by up-regulation of chaperons and enzymes increasing the fold efficiency. Only if the induced stress is unsolvable or passes a certain threshold, the UPR promotes apoptosis [28, 31]. Strikingly, we could measure a significant up-regulation of two heat shock proteins HSPA5 and HSPH1 both promoting cell survival [32, 33]. Moreover, this observation is in line with a study that showed that HSPA5 was increased in AD temporal cortex and hippocampus, determined by Western blot analysis [15]. On the other hand, BAX and AMFR were significantly down-regulated. Several studies have assigned BAX activation as mediator of cell death [34–37] and communicator between the ER and the mitochondria. BAX/BAK double knockout cells revealed no caspase activation following ER stress, indicating that these are required mediators for the communication between the two organelles [34]. Autocrine motility factor receptor (AMFR) is a multifaceted ubiquitin ligase that integrates a unique protein degradation pathway from the endoplasmic reticulum [38]. Moreover, AMFR was also shown to be a mitochondrial-associated ER membrane (MAM)-enriched protein [39, 40] regulating relative positioning of ER and mitochondria by calcium changes [41]. Furthermore, no cell death was measured which supports the assumption that APP cells seem to be able to positively feedback control the ER stress induced by the presence of Aβ. Still, mitochondrial function was dysregulated in basal condition of APP/Aβ-overexpressing cells displayed by decreased ATP level and slightly hyperpolarized mitochondrial membrane potential. The impairment of ATP and MMP in APP/Aβ-overexpressing cells was also previously described in a study of our lab on the identical cell lines [42] and in APP-transfected PC12 cells, another cellular model mimicking Aβ pathology. In this study, the authors suggested that the hyperpolarization may be due to increased nitric oxide levels [43], knowing that Aβ-secretion was in similar low nanomolar range [21, 43] in cells of the two compared studies. Still, it is not clear weather mitochondrial dysfunction is the cause or the consequence of the induced ER stress by Aβ. However, the ER-mitochondrial crosstalk is certainly involved in Aβ-induced apoptosis, since amyloid β-induced ER stress is enhanced under mitochondrial dysfunction conditions.

P301L tau-overexpressing cells also showed an activated UPR in basal condition, as PERK was significantly up-regulated, but the number of up-/down-regulated genes was lower than in APP cells and also the fold change did not increase as significant as in APP cells. Though, it must be considered that P301L cells were compared to WT Tau cells. WT Tau and P301L cells show similar tau expression levels [22, 23]; however, the P301L mutation is required for abnormal tau hyperphosphorylation and filament formation and is found in a familial form of frontotemporal dementia with parkinsonism linked chromosome 17 (FTDP-17) [23]. Of note, WT Tau showed no active UPR compared to Mock cells, so WT Tau cells were considered as control group for P301L cells, to show the effect of the tau mutation per se. Still, P301L-overexpressing cells seemed also to overcome the stress by feedback control of the UPR. Namely, PCNA, ERO1A, and ERO1B, which are all involved in pro-cell survival pathways, were significantly up-regulated. Proliferating cell nuclear antigen (PCNA) was shown to be an essential cofactor for DNA replication and repair [44, 45], whereas endoplasmic reticulum oxidoreductin 1 alpha/beta (ERO1A/B) contribute to the survival of the cell by driving oxidative protein folding [46].

Furthermore, P301L cells seem to differently impair mitochondrial bioenergetics and metabolic activity in basal condition. In line with findings of our lab [42], P301L cells showed decreased mitochondrial membrane potential, paralleled by a drop of ATP level. Several studies already showed that NFTs block mitochondrial transport, which results in energy deprivation and oxidative stress [25, 27]. Although mitochondrial function was dysregulated, the overexpression of P301L-tau did not induce cell death, as no LDH release was detected.

Taken together, APP/Aβ and mutant tau-overexpressing cells display both an activation of the UPR in basal condition by up-/down-regulating genes involved in both pro-cell-survival and pro-cell-apoptosis pathways. However, the two cell types seem to be able to feedback control the induced ER stress by an adaptive response, since no cell death was measured in basal conditions. Furthermore, they both differently impair mitochondrial function, which could be explained by the fact that APP/Aβ and mutant tau target mitochondria differently [47]. The activated UPR and the dysregulation of mitochondrial function are likely to be connected; however, it is not clear whether ER stress is the cause or the consequence of mitochondrial dysregulation.

Acute ER stress by thapsigargin-induced activation of the UPR in all four cell types. Thapsigargin is a commonly used ER stressor which irreversibly inhibits the sarcoplasmic/endoplasmic reticulum Ca^2+^ -ATPase and was shown to induce the UPR in different mammalian cells [28, 48]. However, we observed that APP-overexpressing cells were more sensitive to the induced ER stress than the Mock cell, since many genes were significantly up-/down-regulated when compared to Th-treated Mock cells. Interestingly, the pro-survival gene HSPA5 which was up-regulated in the basal condition was significantly down-regulated after the induced ER stress. On the other hand, pro-apoptotic genes such as BAX and AMFR which were down-regulated in basal condition were significantly up-regulated after the induced ER stress. Taken together, APP-overexpressing cells seem to handle the cellular stress by the overexpression of APP by feedback control of the activated UPR and up-regulate genes involved in pro-cell survival pathways, whereas an additionally induced ER stress by thapsigargin can no longer be counteracted by the cell, favouring up-regulation of genes involved in pro-apoptotic pathways. In line with the output of the experiment on gene expression, we could measure a significant increase of LDH level in Th-treated APP-overexpressing cells, indicating cellular death. Furthermore, the induced ER stress seemed also to disturb mitochondrial function. Even if the ER stress induced an increase of the ATP level and the mitochondrial membrane potential in APP and Mock cells compared to their basal condition in the two individual cell lines, ATP levels were reduced in Th-treated APP-overexpressing cells compared to Th-treated Mock cells. Indeed, the decreased mitochondrial function might be the consequence of reduced cell viability caused by the Th-induced ER stress; however, it also reveals the higher vulnerability of APP-overexpressing cells.

Acute Th treatment on P301L cells showed no UPR activation and no significant dysregulation of mitochondrial function when compared to Th-treated WT Tau cells. Interestingly, when we compared Th-treated P301L and WT Tau cells to Th-treated Mock cells, we observed a significant activation of the UPR and an identical strong increase of the same genes. While in basal condition, WT Tau cells show no UPR and P301L have a significant activation of the UPR, and both cell types identically turn on the UPR after acute Th treatment. In basal condition, P301L cells express high level of phospho-tau, whereas WT Tau cells show hardly any expression. Interestingly, when cells were treated with thapsigargin, both WT Tau and P301L cells displayed similar high level of phospho-tau expression. Therefore, we suggest a direct functional connection between UPR activation and tau phosphorylation. Indeed, Th was shown to induce phosphorylation of tau in several studies [20, 49–51]. In vitro and in vivo data on cultured cells (HEK293 and SH-SY5Y cells) and rat brain demonstrate that thapsigargin treatment is accompanied by increased tau phosphorylation [49, 50]. These findings further advert that phosphorylation of tau and ER stress could be induced by each other to form a vicious cycle, thereby accelerating AD-like neurodegeneration. Moreover, both WT and mutant tau cells showed decreased ATP level and a depolarized mitochondrial membrane potential, indicating that tau-overexpressing cells are more vulnerable to thapsigargin-induced ER stress than Mock cells. This finding further supports the close communication between mitochondria and the ER during apoptosis in AD.

Our experiment could further confirm that overexpression of the two AD hallmark proteins APP/Aβ and tau induces ER stress as shown by the activation of the UPR and dysregulation of mitochondrial function. Strikingly, we could show that both cell types overcome this ER stress by a positive feedback control of the UPR by up-regulating pro-survival genes, whereas pro-apoptotic genes are down-regulated. However, when the ER stress is increased, both cell types can no longer feedback control the ER stress and increase gene expression favouring apoptotic pathways, displayed by cell death in APP-overexpressing cells already after 3 h. Interestingly, we also measured an identical response of the activated UPR genes in WT Tau cell and P301L cells, as and identical expression of phospho-tau, indicating a direct functional connection between thapsigargin-induced UPR activation and phosphorylation of tau.

This study further supports the close communication between mitochondria and the ER during apoptosis in AD. However, further experiments focusing on the role of this interaction and the underlying mechanisms involved in the ER—and mitochondrial stress response in AD cellular model would be of great interest to develop potential new drugs for the prevention and/or treatment of neurological diseases including AD.

### Electronic supplementary material

Below is the link to the electronic supplementary material.

**Supplementary Figure 1: Fold change of basal WT Tau cells compared to Mock cells.** (A) Basal fold-change regulation of UPR genes of WT Tau cells compared to non-treated Mock cells. Values were calculated based on a Student’s *t* test of the replicate 2^(-Delta Ct) values for each gene, and p < 0.05 were considered significant. Values represent the mean ± SEM (n=18–60 replicates of 3–5 independent experiments) and were normalized to the basal (non-treated) condition of each cell type, respectively (=100%). Statistical analysis was performed using One-Way ANOVA followed by Turkey’s Multiple Comparison Test: *** P<0.001

**Supplementary Figure 2: ATP and MMP level in (A, C) differentiated APP and (B, D) differentiated P301L cells compared to differentiated Mock and WT Tau cells, respectively.** ATP and MMP levels were measured in (A, B) basal condition and (C, D) after 3 h thapsigargin treatment. Values represent the mean ± SEM (n = 12–18 replicates of three independent experiments) and were normalized to 100 % of (A, C) differentiated Mock cells or (B, D) differentiated WT Tau cells. Student unpaired *t* test, *P < 0.05; **P < 0.01; ***P < 0.001

**Supplementary Figure 3: Analysis of fluorescence intensity of phospho-tau (AT8) in WT Tau and P301L cells in basal condition and after Th = thapsigargin (500 nM, 3 h) or OA = okadaic acid (100 nM, 3h) treatment.** Values represent the mean ± SEM fluorescence relative to total area of cell (n= 12–36 cells of 3 independent experiments). Statistical analysis was performed using One-Way ANOVA followed by Turkey’s Multiple Comparison Test. ImageJ software was used to quantify intensity of phospho-tau protein

**Supplementary Table 1: Fold change of basal APP cells vs. basal Mock cells and acute Th-treated APP cells vs. acute Th-treated Mock cells.** Fold-change values greater than 2 are indicated in red; fold-change values less than 0.5 are indicated in blue. The *p* values are calculated based on a Student’s *t* test of the replicate 2^(-Delta CT) values for each gene in the control group (Mock cells) and treatment group (APP cells), and *p* values less than 0.05 are indicated in red

**Supplementary Table 2: Fold change of basal WT Tau cells vs. basal Mock cells and acute Th-treated APP cells vs. acute Th-treated Mock cells.** Fold-change values greater than 2 are indicated in red. The p values are calculated based on a Student’s *t* test of the replicate $2^{{ - \Delta {\text{Ct}}}}$ values for each gene in the control group (Mock cells) and treatment group (WT Tau cells), and p values less than 0.05 are indicated in red

**Supplementary Table 3: Fold change of basal P301L cells vs. basal WT Tau cells and acute Th-treated P301L cells vs. acute Th-treated WT Tau and Mock cells.** Fold-change values greater than 2 are indicated in red; fold-change values less than 0.5 are indicated in blue. The *p* values are calculated based on a Student’s *t* test of the replicate $2^{{ - \Delta {\text{Ct}}}}$ values for each gene in the control group (WT Tau and Mock cells) and treatment group (P301L cells), and *p* values less than 0.05 are indicated in red

**Supplementary Table 4: 84 UPR genes classified by pathway involved**

## Abbreviations

Aβ: Amyloid-β peptide
AD: Alzheimer’s disease
APP: Amyloid-β precursor protein
ER: Endoplasmic reticulum
LDH: Lactate dehydrogenase
MMP: Mitochondrial membrane potential
P301L: Tau mutation
PERK: Protein kinase RNA-like endoplasmic reticulum kinase
Th: Thapsigargin
UPR: Unfolded protein response
WT Tau: Wild-type tau
