# Supplementary figures and images for: Link between the unfolded protein response and dysregulation of mitochondrial bioenergetics in Alzheimer’s disease
Source: Cell Mol Life Sci. 2019 Jan 25;76(7):1419–31. doi: 10.1007/s00018-019-03009-4 (PMC6420888; doi:10.1007/s00018-019-03009-4)

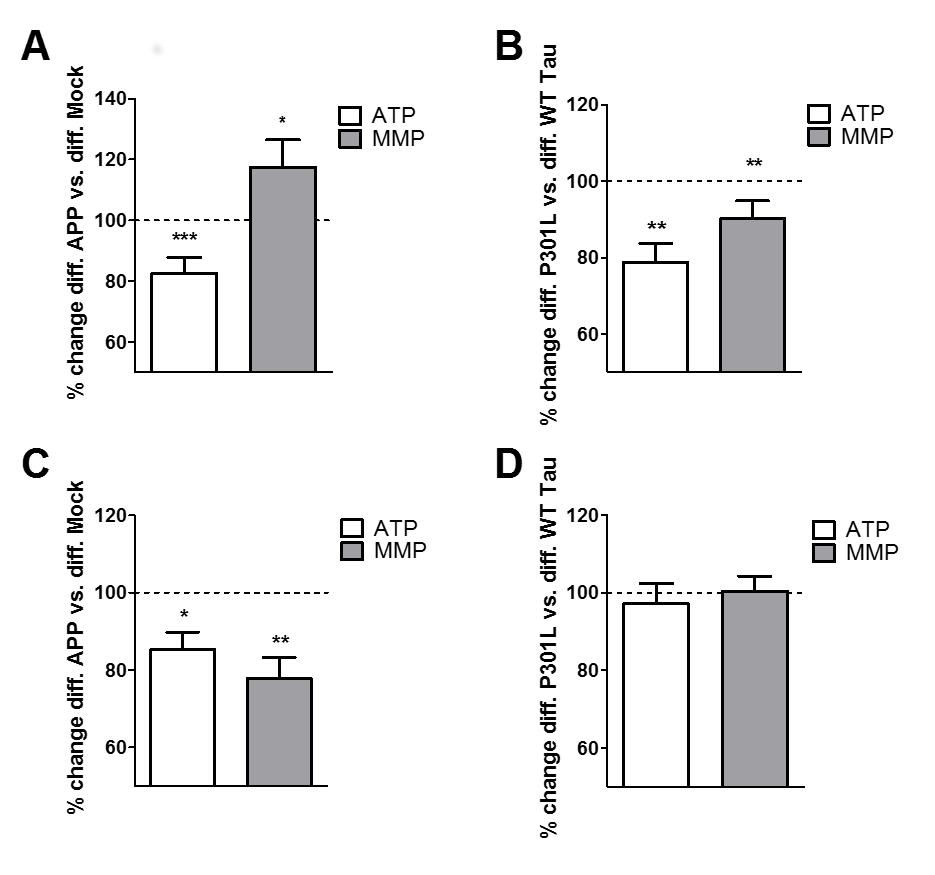

Supplement: Supplementary file 2 — Supplementary Figure 2: ATP and MMP level in (A, C) differentiated APP and (B, D) differentiated P301L cells compared to differentiated Mock and WT Tau cells, respectively. ATP and MMP levels were measured in (A, B) basal condition and (C, D) after 3 h thapsigargin treatment. Values represent the mean ± SEM (n = 12–18 replicates of three independent experiments) and were normalized to 100 % of (A, C) differentiated Mock cells or (B, D) differentiated WT Tau cells. Student unpaired t test, *P < 0.05; **P < 0.01; ***P < 0.001 [file 18_2019_3009_MOESM2_ESM.tif]
