# Supplementary material for: Link between the unfolded protein response and dysregulation of mitochondrial bioenergetics in Alzheimer’s disease
Source: Cell Mol Life Sci. 2019 Jan 25;76(7):1419–31. doi: 10.1007/s00018-019-03009-4 (PMC6420888; doi:10.1007/s00018-019-03009-4)
Supplement: Supplementary file 7 — Supplementary Table 4: 84 UPR genes classified by pathway involved [file 18_2019_3009_MOESM7_ESM.docx]

**Supplementary Table 4**
